# Case Report: Unveiling the enigma: a rare male neonatal case of MIRAGE syndrome with female external genital presentation and literature review

**DOI:** 10.3389/fped.2026.1808088

**Published:** 2026-06-12

**Authors:** Shuyan Li, Fangjian Gao, Xiaojuan Lin, Dongmei Wang, Shuangquan Gao, Yu Ding, Jianwu Qiu

**Affiliations:** 1 Guangdong Medical University, Zhanjiang, Guangdong, China; 2 Department of Neonatology, YueBei People’s Hospital, Guangdong Medical University, Shaoguan, Guangdong, China; 3 Guangdong Province Critical Newborn Rescue Center (North Guangdong), Shaoguan, Guangdong, China; 4 Department of Pathology, YueBei People’s Hospital, Guangdong Medical University, Shaoguan, Guangdong, China

**Keywords:** disorders of sex development, growth restriction, MIRAGE syndrome, placental insufficiency, *SAMD9* gene

## Abstract

**Background:**

MIRAGE syndrome is a severe congenital disease affecting multiple systems, caused by functional variants in the *SAMD9* gene. It is characterized by myelodysplasia, infections, growth restriction, adrenal hypoplasia, genital phenotypes, and enteropathy. There are few reports of neonatal MIRAGE syndrome. This study presents a rare case of 46,XY karyotype with distinct female external genitalia phenotype and provides a comprehensive literature review of infants under 1 year of age diagnosed with MIRAGE syndrome caused by *SAMD9* gene mutations.

**Case presentation:**

This article reports a sporadic case of neonatal MIRAGE syndrome confirmed by genetic diagnosis. The patient had a 46, XY karyotype and presented predominantly with female external genitalia, along with preterm birth, respiratory distress, growth restriction, recurrent infections, skin pigmentation, feeding difficulties, thrombocytopenia, anemia, and other manifestations.

**Conclusion:**

In clinical practice, when encountering newborns with unexplained premature birth, growth restriction, thrombocytopenia, recurrent infections, and a karyotype of 46, XY but with female or ambiguous external genitalia, clinicians can, based on the experience from this case, differentiate from MIRAGE syndrome and may further perform genetic testing to clarify the etiology.

## 1 Introduction

MIRAGE syndrome (MIM 617053) is a severe multisystem congenital disorder first reported by Narumi S in 2016 (1). It is characterized by myelodysplasia, infection, growth restriction, adrenal hypoplasia, genital phenotypes, and enteropathy,hence the acronym MIRAGE. The disease is caused by functional variants in the growth repressor sterile alpha domain containing 9 (*SAMD9*) located on the arm of chromosome 7 (7q21.2) (2). Patients with this condition often died before age 2 years due to the invasive infection (3).

This article reports a rare neonatal case of MIRAGE syndrome confirmed by genetic analysis. During pregnancy, the fetus presented with intrauterine growth restriction, and intrauterine distress was observed at birth. The key clinical features after birth included genital abnormalities, prematurity, low birth weight, significant skin hyperpigmentation, respiratory distress, recurrent infections, thrombocytopenia, anemia, severe feeding difficulties, patent ductus arteriosus and patent foramen ovale, delayed white matter development, and ventriculomegaly. There are few documented cases of neonatal MIRAGE syndrome both domestically and internationally. This study also conducted a retrospective analysis of publicly reported cases of MIRAGE syndrome in infants under one year of age, seeking to enhance clinical recognition of this disease and mitigate the risks of misdiagnosis and missed diagnosis.

## 2 Case presentation

### 2.1 Clinical data

The infant was the second-born child in the family, with a gestational age of 33 weeks and 4 days, and was admitted to the hospital due to “shallow and rapid breathing with moaning for 14 min after birth.” The infant was delivered by cesarean section due to “fetal distress *in utero*; gestational diabetes mellitus” in the mother, with grade III amniotic fluid turbidity; the Apgar score was normal(1 min: 9 points, with 1 point deducted for skin color; 5 min and 10 min: both 10 points), birth weight was 1490 g (below the 10th percentile for average birth weight of the same gestational age), and body length was 41 cm (between the 10th and 25th percentiles). After birth, the infant's breathing remains shallow and rapid, with persistent frothing at the mouth, accompanied by a distinct moaning-like respiration, transcutaneous oxygen saturation of 88%. The infant was transferred to the neonatal intensive care unit under positive pressure ventilation.

The parents are non-consanguineous with no significant family history of genetic disorders. Initial ultrasound at 13 weeks and 6 days indicated a gestational age measuring only 11 weeks and 5 days. Subsequent intrauterine color Doppler examination at 23 weeks + 6 days revealed a single live fetus, size consistent with approximately 22 + weeks gestation; notably, an echogenic focus was detected within the fetal left ventricle, and the umbilical cord encircled the neck for three loops. A follow-up scan at 31 weeks + 6 days confirmed a single live fetus with overall size equivalent to roughly 30 weeks gestation, again showing the umbilical cord encircling the neck three times. Prenatal level I color Doppler ultrasound at 33 weeks + 3 days revealed the following findings: Biparietal Diameter 85 mm, Head Circumference 294 mm, Abdominal Circumference 249 mm (significantly below the 3rd percentile for gestational age), Femur Length 59 mm, Humerus Length 54 mm. The scan confirmed an intrauterine singleton live pregnancy, showing fetal biometrics corresponding to approximately 32 weeks’ gestation. A nuchal cord with two loops was observed, accompanied by persistently abnormal fetal umbilical artery blood flow indices (reversed end-diastolic flow in the fetal umbilical artery). The fetal ultrasound biophysical profile scored 6 points.

**Figure 1:**
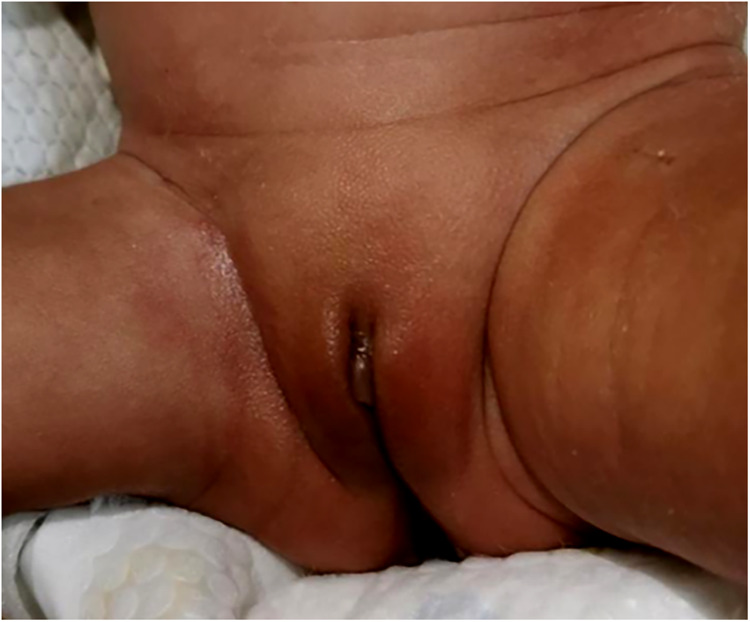
The infant presents with typical female external genitalia. The skin color in the genital area is dark, and the labia majora are well-developed, partially covering the labia minora. The anus is in a normal position and remains patent. The overall appearance of the external genitalia is consistent with that of a healthy female infant.

Upon admission, the infant's physical examination results showed: temperature (T) 36.4 °C, pulse (P) 138 beats/min, respiratory rate (R) 62 breaths/min, blood pressure (BP) 60/38 mmHg. The infant was conscious but sluggish, in a premature state. The skin was uniformly dull. Tachypnea was observed, with diminished breath sounds in both lungs. Heart rate was 138 beats/min, regular rhythm, no murmurs heard in the valve areas. The abdomen was soft and flat, liver and spleen not palpable below the costal margin, bowel sounds normal. Bilateral inguinal areas were palpable with swelling, and the genitalia appeared female (Figure 1). Limb muscle tone was mildly decreased, and neonatal reflexes were not elicited. Assessment indicated a gestational age of 32 weeks. Blood glucose was 1.2 mmol/L at admission.

Upon admission, the infant immediately underwent respiratory support via Non-Invasive Positive Pressure Ventilation(NIPPV),commenced antibiotic therapy (cefotaxime-sulbactam plus penicillin), received intravenous glucose infusion, underwent umbilical venous catheterization, received vitamin K1 administration, and initiated parenteral nutrition.

On the second day after admission, severe thrombocytopenia (platelet count 36 × 10⁹/L) emerged; intravenous immunoglobulin (2 g/kg) was administered. By the fourth day, the platelet count plummeted to 24 × 10⁹/L. Following platelet transfusion therapy, the platelet count rebounded to normal levels.

By the third day of hospitalization, respiratory symptoms showed improvement, and the patient was gradually transitioned to continuous positive airway pressure (CPAP). Respiratory support was successfully discontinued on the sixth day. However, at that time, follow-up infection markers showed an increase compared to previous levels, and cefotaxime and sulbactam anti-infective therapy was continued. On the tenth day of hospitalization, the infant again developed tachypnea and dyspnea. Repeat infection markers showed a significant increase in C-reactive protein (CRP), and chest radiography indicated worsening bilateral pneumonia compared to previous findings. Considering inadequate infection control, anti-infective therapy was intensified by adjusting to meropenem (10-day course) combined with vancomycin (7-day course), leading to further clinical improvement.

On the twelfth day, the blood fungal 1,3-*β*-D glucan test result returned at 87.91 pg/mL, confirming a fungal infection, and fluconazole was promptly administered to initiate antifungal treatment.

On day 36, the infant developed fever, tachypnea, and respiratory distress, accompanied by markedly elevated infection markers. CPAP respiratory support commenced alongside anti-infective therapy with cefoperazone and sulbactam. Due to a suboptimal response, treatment transitioned to meropenem to intensify anti-infective management. Concurrently, hemoglobin concentration measured 86.0 g/L, which returned to baseline following blood transfusion therapy. The infant presented with generalized skin darkening and hyperpigmented nipples, raising suspicion of congenital adrenal hyperplasia (CAH), prompting a second CAH screening.

On day 54 of hospitalization, the hemoglobin concentration dropped to 98.0 g/L, and it returned to normal after another blood transfusion therapy.

By the 57th day of hospitalization, the infant's infection symptoms improved, and follow-up infection markers showed no significant elevation. Antibiotic therapy and oxygen therapy were discontinued. The infant's weight gain during admission was unsatisfactory, with a weight of only 2.54 kg at a corrected gestational age of 42 weeks. The infant was hospitalized for 58 days. After the diagnosis of MIRAGE syndrome was confirmed, the family requested the infant's discharge from the hospital. At the time of discharge, the infant had feeding difficulties and was unable to suckle independently, exhibiting poor sucking ability and uncoordinated swallowing function. This prompted the family to request discharge with a gastric tube.

Five days post-discharge, the infant suddenly developed facial cyanosis and suffered cardiac and respiratory arrest at home. The infant was urgently transported to the emergency department of our hospital. The electrocardiogram indicated cardiac arrest, and the infant was pronounced dead.

### 2.2 Auxiliary examination

#### 2.2.1 General laboratory tests and examinations

The complete blood count (CBC) and Infection Markers are shown in Table 1.

**Table 1:** Monitoring of blood white cells, platelets, and infection markers during hospitalization.

Content Items	1d	2d	4d	6d	10d	12d	17d	36d	38d	41d	46d	54d	57d
WBC × 10^9^/L	14.82	11.78	7.03	11.20	10.09	9.77	10.66	11.25	15.61	10.46	13.38	8.45	9.8
HGB g/L	174	167	155	141	126	132	126	86	157	143	135.0	98.0	141
PLT × 10^9^/L	66	36	24	123	91	90	117	160	119	101	103	140	122
CRP mg/L	-	3.66	-	11.37	104.80	40.26	4.03	21.32	61.81	8.69	-	-	-
PCT ng/mL	-	0.641	-	1.164	-	0.718	0.236	2.210	4.763	0.607	0.254	0.671	-

WBC, white blood cell count; HGB, hemoglobin concentration; PLT, platelets; CRP, C - reactive protein; PCT, procalcitonin.

Multiple serum electrolyte tests during hospitalization showed no significant abnormalities in sodium and potassium ions, and multiple thyroid function tests indicated elevated thyroid-stimulating hormone (TSH), with free triiodothyronine (T3) and free thyroxine (T4) within normal ranges.

A second screening for CAH showed: 17-hydroxyprogesterone level <4.26 nmol/L, androstenedione level 4.38 nmol/L, cortisol level 111.11 nmol/L, 11-deoxycortisol level 19.99 nmol/L, 21-deoxycortisol level <2.35 nmol/L, and the (17-hydroxyprogesterone + androstenedione)/cortisol ratio was 0.08.

Sex hormone levels: Progesterone: 11.100 nmol/L, Testosterone: 4.27 nmol/L, Estradiol: <5.00 pg/mL, Follicle-stimulating hormone(FSH): 13.00 mIU/mL, Luteinizing hormone(LH): 29.60 mIU/mL, Prolactin: 165.00 ng/mL.

Both the initial and follow-up hearing screenings, utilizing Otoacoustic Emissions and Auditory Brainstem Response tests, revealed that neither ear passed.

An amplitude-integrated electroencephalogram was mildly abnormal.

#### 2.2.2 Imaging examination

Chest radiography on day 1 revealed neonatal respiratory distress syndrome; on day 10, bilateral pneumonia was noted, with increased pulmonary inflammation compared to previous images; by day 36, bilateral bronchopneumonia was diagnosed; and on day 41, the bronchopneumonia showed more pronounced lesions.

Echocardiography indicated a patent ductus arteriosus and patent foramen ovale. Pediatric cranial ultrasound detected widening of the right lateral ventricle. Superficial organ ultrasound identified an oval hypoechoic focus in each inguinal region, with clear boundaries, moving between the abdominal cavity and the inguinal region with respiration, and internal echoes similar to testicular parenchymal echoes. No uterine or adnexal echoes were observed. Routine adrenal color Doppler ultrasound examination showed: no significant abnormalities in either adrenal gland.

Cranial magnetic resonance imaging (MR) revealed poor development of brain white matter.

#### 2.2.3 Histological analysis of the placenta

**Figure 2:**
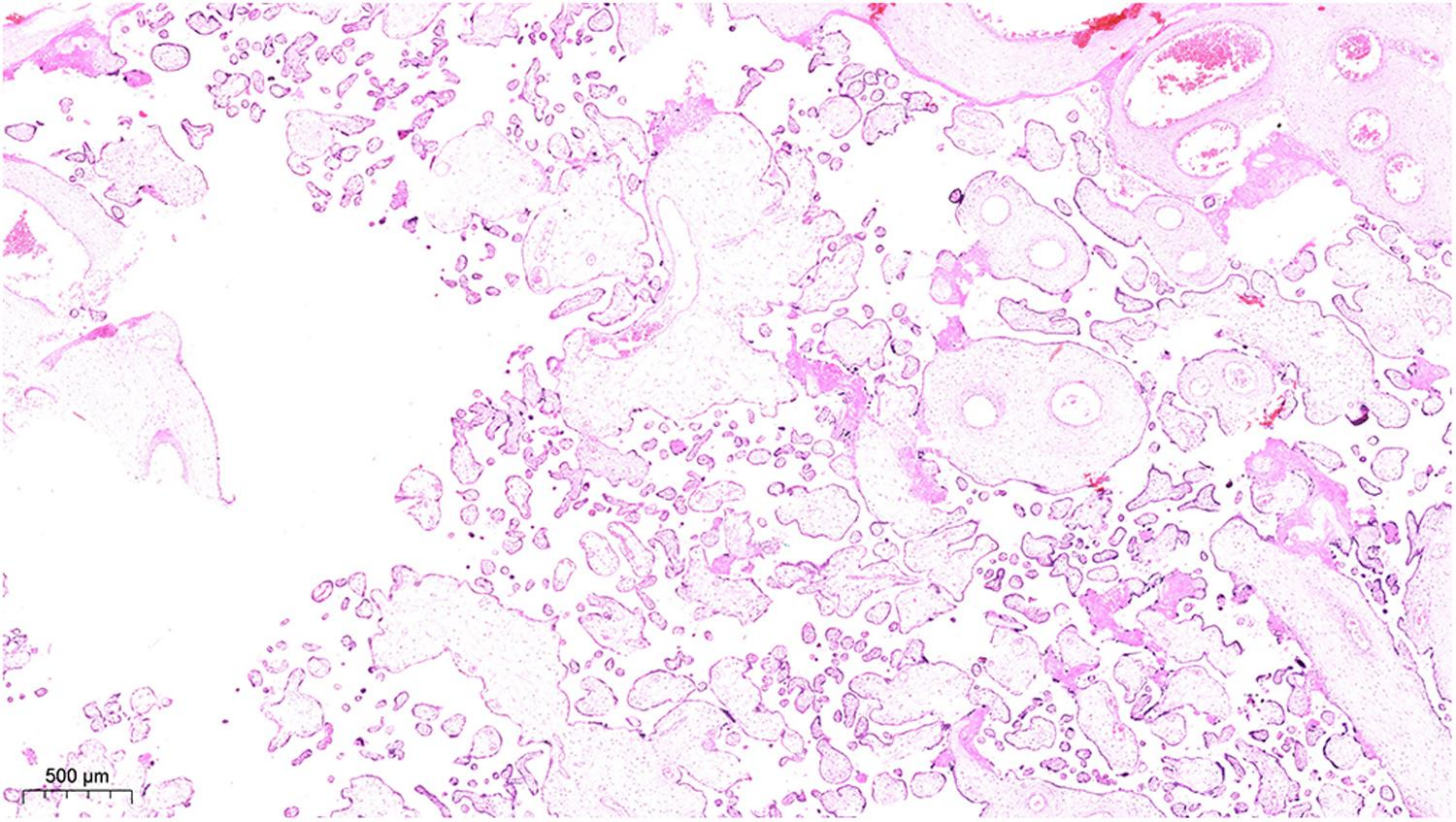
The placental tissue of the infant. The terminal villi are hypoplastic and reduced in number, with significant dilation of the intervillous spaces.

To reveal the mechanism of small for gestational age (SGA), we performed a histological examination of the patient's placental tissue. Histological evaluation of the patient's placental tissue revealed hypoplasia of the distal villous trees and enlarged intervillous spaces (Figure 2). The placental findings in this case are consistent with those reported in two previously published cases carrying the p.Arg459Gln and p.Phe1017Val variants, characterized by hypoplasia of the distal villous trees and enlarged intervillous spaces (1, 4).

#### 2.2.4 Gene result

Following birth, Y chromosome gene signals emerged during routine newborn screening for common genetic disorders, triggering laboratory gender quality control failure. Consequently, chromosomal karyotype testing was advised. Karyotype analysis revealed a 46,XY result, with no abnormal chromosomes detected at the 320-400 band resolution. The infant was strongly suspected of having 46,XY DSD(Disorders of Sex Development), presenting with concurrent multisystem manifestations including growth restriction, infection, hyperpigmentation, feeding difficulties, thrombocytopenia, and anemia, prompting further investigation with whole exome sequencing.

**Figure 3:**
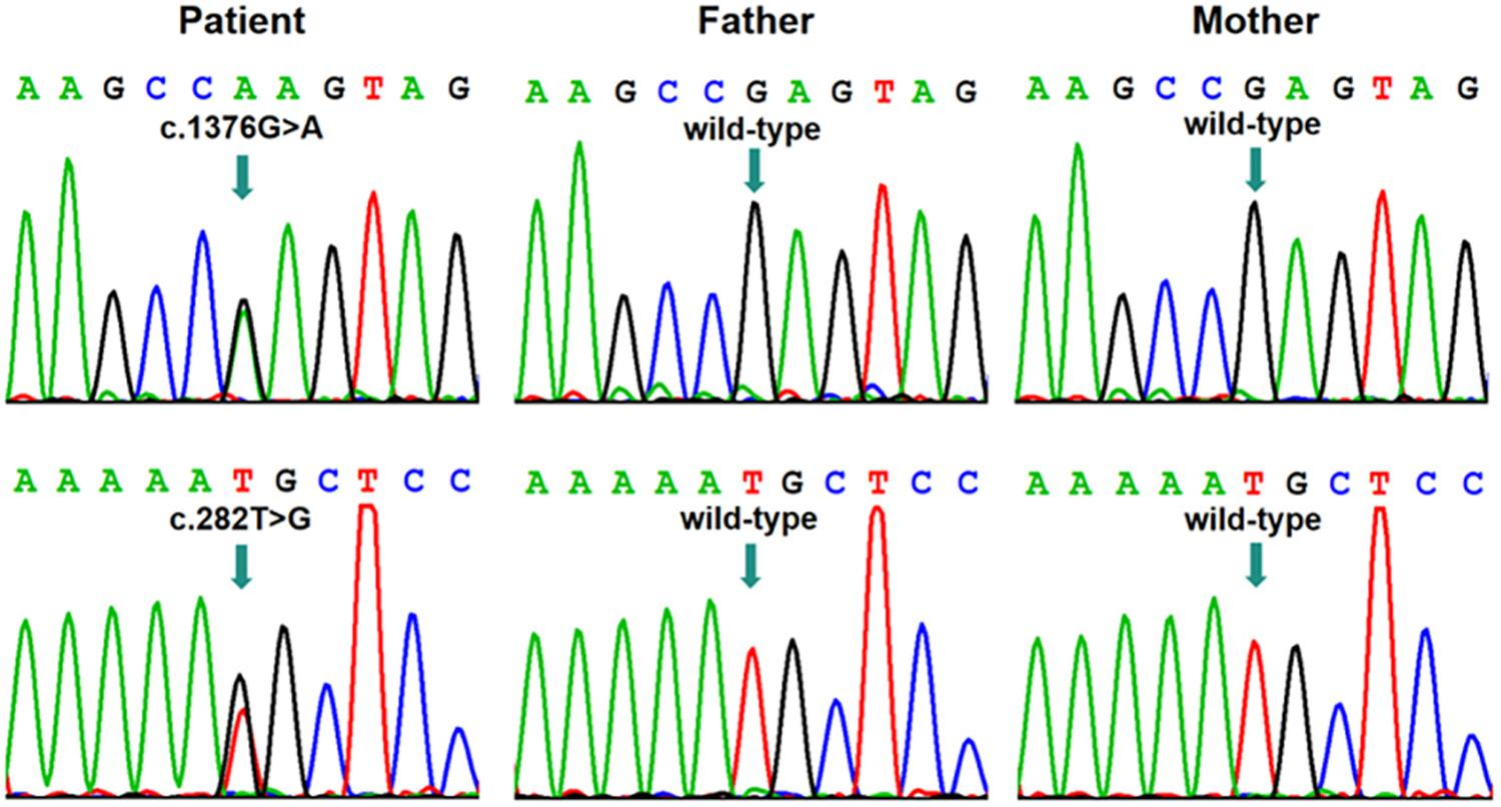
Sanger sequencing results. Genetic analysis indicates the presence of heterozygous *SAMD9* variants c.1376G > A (p.Arg459Gln) and c.282T > G (p.Asn94Lys) in the infant, with no mutations detected in the parents. The mutation sites are indicated by arrows.

Whole exome sequencing identified two heterozygous variants in the *SAMD9* gene: c.1376G > A (p.Arg459Gln) and c.282T > G (p.Asn94Lys). Upon validation by Sanger sequencing, both parents were confirmed to be wild-type (Figure 3). The parents do not carry these two variants, and these two variants are *de novo*. The c.1376G > A (p.Arg459Gln) variant is a missense mutation, resulting in the substitution of arginine (Arg) at position 459 by glutamine (Gln). This variant is a previously reported pathogenic variant (1). The c.282T > G (p.Asn94Lys) variant is a missense mutation, leading to the replacement of asparagine (Asn) at position 94 with lysine (Lys). This variant has not been previously reported in the literature and is considered a novel mutation. As predicted by software such as SIFT and PolyPhen - 2, the scores are 0.527 and 0.005, respectively, indicating that it is regarded as a tolerable variant. According to the American College of Medical Genetics and Genomics (ACMG) guidelines, the site is a VUS (Variant of Uncertain Significance).

## 3 Literature review

### 3.1 Methods

We performed a rigorous literature search spanning databases such as PubMed, Web of Science, and key Chinese resources (China National Knowledge Infrastructure, Wanfang Data Knowledge Service Platform, and VIP Information Journal Database). Our search utilized the terms “MIRAGE Syndrome” OR “*SAMD9*” and encompassed all records from each database's inception until November 2025. This search identified 70 neonates and infants (under 1 year old) with *SAMD9* gene mutation-induced MIRAGE syndrome, collectively documented across 50 published studies (1, 2, 4–51).

### 3.2 Results

Overall, the analysis included data from 70 patients harboring SAMD9 gene variants (refer to Supplementary Table S2). Geographically, the cases spanned diverse regions, with the majority documented in Japan (34%, 20/59), the United States (19%, 11/59), and China (14%, 8/59); collectively, these nations accounted for roughly two-thirds of all reported instances.

In terms of perinatal and birth history, the mean gestational age was 32.36 ± 3.16 weeks (range: 26–39 weeks). Preterm birth—defined as delivery before 37 weeks—was strikingly common, affecting 92% (47/51) of the infants. The mean birth weight was 1330.16 ± 451.56 g (range: 900–2790 g), with a substantial majority (98%, 42/43) classified as low birth weight infants (<2500 g). Growth restriction was nearly ubiquitous, identified prenatally as intrauterine growth restriction in 96% (52/54) of cases and demonstrated postnatally in 98% (44/45).

Clinically, abnormalities of the external genitalia manifested in 75% (45/60) of patients. Primary presentations included cryptorchidism (38%, 17/45), hypospadias (44%, 20/45), and ambiguous genitalia. Notably, among those with genital anomalies, 31% (14/45) possessed a 46,XY karyotype yet presented with female or ambiguous external genitalia. Beyond these, other prominent clinical features encompassed frequent infections (95%, 60/63), enteropathy (94%, 48/51), and adrenal insufficiency (78%, 47/60). Within the latter group, 17 cases were linked to adrenal hypoplasia, 4 to adrenal agenesis, and 1 to adrenal atrophy. Additionally, two documented cases presented adrenal hypoplasia without recorded adrenal insufficiency.

Hematological alterations stood as a hallmark of the disease. Thrombocytopenia emerged as the most prevalent manifestation, affecting 93% (54/58) of patients, followed by anemia in 77% (37/48). Myelodysplasia was identified in 49% (19/39) of patients, with 9 of these cases having progressed to myelodysplastic syndrome (MDS).

Through genetic analysis, a total of 46 distinct mutation sites were identified among 70 patients carrying *SAMD9* gene variants. The variant c.3878G > A was the most frequent (13%, 6/46), followed by c.1376G > A and c.2471G > A (11% each, 5/46), representing recurrent hotspot mutations. The remaining mutation sites accounted for approximately 65% of the total.

At the final follow-up, the overall mortality rate reached 45% (30 of 67 patients). Primary causes of death comprised infection (4 cases), multiorgan failure (4 cases), and myelodysplastic syndromes (3 cases). The median age at death was 1.09 years (Interquartile RangeInterquartile Range, IQR: 0.40–2.00).

## 4 Discussion and conclusion

MIRAGE syndrome is caused by pathogenic variants in the *SAMD9* gene and is an autosomal dominant genetic disorder. It is an extremely rare syndrome with a high mortality rate and a prevalence of less than 1 in 1,000,000 (3). *SAMD9* (sterile *α* motif domain-containing protein 9) is a single exon gene that encodes a 1,589-amino acid protein and is located on the long arm of chromosome 7 (7q21.2) in humans. The gene is widely expressed in the adrenal glands, colon, bone marrow, liver, immune system, lungs, and testes, which explains the diverse clinical manifestations of MIRAGE syndrome. The molecular function of *SAMD9* remains incompletely understood, but the protein is known to localize to the cytoplasm and participate in viral host defense, cell proliferation, endosome fusion, and tumor suppression. As a congenital growth inhibitor, *SAMD9* modulates growth factor signaling pathways; gain-of-function variants enhance this activity, resulting in severe multisystem growth restriction (10, 52, 53).

Notably, pathogenic variants in *SAMD9* are also associated with other distinct clinical entities, including Normophosphatemic Familial Tumoral Calcinosis (NFTC, MIM 610455) (54), and Monosomy 7 myelodysplasia and leukemia syndrome-2 (M7MLS2, MIM 619041) (55). Despite this phenotypic diversity, no clear correlation exists between specific *SAMD9* genotypes and clinical outcomes. However, variants causing MIRAGE syndrome tend to cluster in specific functional domains of the SAMD9 protein, particularly the P-loop NTPase domain (52). Consistent with this observation, certain variants in specific residues, such as Arg459 and Arg1293, are consistently associated with MIRAGE syndrome according to the literature (3).

MIRAGE syndrome is more common among preterm infants, most of whom are born via iatrogenic preterm delivery by cesarean section due to fetal distress. The mean gestational age is typically less than 31 weeks, and the condition may be associated with intrauterine growth restriction. At birth, most affected infants exhibit signs of fetal distress. Growth restriction is observed in 98% of patients, characterized by being small for gestational age at birth followed by Postnatal growth restriction. Myelodysplasia affects 83% of patients, which may present as thrombocytopenia and anemia during the neonatal period, and may also progress to monosomy 7 myelodysplastic syndrome. Recurrent and invasive infections occur in 96% of children. Adrenal hypoplasia is present in 71% of children, with cutaneous hyperpigmentation noted during the neonatal period. Genital phenotype abnormalities are observed in 97% of patients, including hypospadias, microphallus, and undescended testes in affected males, as well as ovarian dysgenesis in females. Gastrointestinal dysfunction, characterized by chronic watery diarrhea and achalasia cardia, occurs in 83% of children (3, 56). Although the above are common features, other additional clinical manifestations are expanding, including achalasia or gastroesophageal reflux, recurrent intussusception, renal features such as focal segmental glomerulosclerosis, and neurological manifestations such as microcephaly, hydrocephalus, and white matter abnormalities. In addition, there can also be congenital heart diseases such as patent ductus arteriosus and ventricular septal defect, joint deformities, hearing impairment, and other manifestations (27, 47, 52).

The infant presented with fetal distress, growth restriction, thrombocytopenia, anemia, recurrent infections, skin pigmentation, feeding difficulties, patent ductus arteriosus, widened lateral ventricles, cerebral white matter dysplasia, and failed hearing screening, with histological examination of the placental tissue revealing hypoplasia of the distal villous trees and enlarged intervillous spaces. Prenatal ultrasound at 33 weeks and 3 days showed reversed end-diastolic flow in the umbilical artery, indicative of placental insufficiency (57). Furthermore, this ultrasound revealed an abdominal circumference of 249 mm, which fell below the 3rd percentile for gestational age according to international fetal growth standards (58).Therefore, based on guideline consensus, this establishes the diagnosis of fetal growth restriction (FGR) (59). Left ventricular echogenic focus is a common soft marker in prenatal ultrasound examinations and can serve as an early manifestation of multisystem developmental abnormalities. However, current literature has not yet listed left ventricular echogenic focus as a typical prenatal feature of MIRAGE syndrome.

The literature indicates that evaluation of placental tissues from patients with *SAMD9* variants shows poorly developed distal villous trees with widening of the intervillous space, suggesting abnormal development. The higher expression level of SAMD9 in the placenta during later pregnancy may be a contributing factor to poor growth and fetal distress in children with MIRAGE syndrome, and also a reason for the premature delivery of these infants (52). Of note, in MIRAGE syndrome, the pathogenesis of SGA and genital anomalies is multifactorial. On one hand, pathogenic mutations in the SAMD9 protein exert potent cell growth inhibitory effects, directly leading to global growth restriction and testicular dysplasia. On the other hand, *SAMD9* variants result in placental villous hypoplasia, contributing to placental insufficiency characterized by inadequate blood supply and diminished hCG stimulation. Therefore, these two mechanisms may be related to SGA and genital abnormalities in children with phantom syndrome (4).

Disorders of sex development (DSD) are defined as congenital conditions in which chromosomal, gonadal, or phenotypic sex is atypical. Clinical presentations encompass hypospadias, ambiguous genitalia, and complete XX or XY sex reversal. The proband had a karyotype of 46,XY but presented with female external genitalia characteristics. Echogenic structures resembling testicular parenchyma were observed in the bilateral inguinal regions, with no uterus or adnexa visualized. Testosterone, LH, and FSH levels were elevated, supporting a diagnosis of 46,XY DSD. However, whole exome sequencing failed to identify pathogenic variants in genes commonly linked to DSD, including those involved in complete or partial gonadal dysgenesis, androgen insensitivity syndrome, or Denys-Drash/Frasier syndrome (60). Beyond the DSD phenotype, the infant also displayed skin hyperpigmentation. Initial evaluations for CAH—including electrolyte tests and bilateral adrenal ultrasonography—did not support this diagnosis. Furthermore, whole exome sequencing revealed no disease-causing mutations in genes associated with CAH, such as common variants in the *CYP21A2* gene (61). In light of recurrent infections, thrombocytopenia, anemia, and the detection of two mutation sites in the *SAMD9* gene via genetic testing, we are inclined to interpret the infant's DSD and skin hyperpigmentation as manifestations of MIRAGE syndrome.

This case reports two *de novo* heterozygous mutations in the *SAMD9* gene: c.1376G > A (p.Arg459Gln) and c.282T > G (p.Asn94Lys). Regarding the c.1376G > A (p.Arg459Gln) mutation: This variant constitutes a previously reported pathogenic hotspot mutation, specifically located within the highly conserved P-loop NTPase domain. Functional experiments have confirmed that this mutation exerts an inhibitory effect on cell growth (1). Consistent with previous reports (Supplementary Table S2: cases 11, 16, 34, 36, 37), patients carrying this variant typically present with prematurity, low birth weight, growth restriction, recurrent infections, and adrenal insufficiency. Those with a 46,XY karyotype exhibit female or ambiguous genitalia. Thrombocytopenia and anemia were universal findings, with no evidence of myelodysplasia. This variant is linked to a severe phenotype; among six pediatric cases (including the present one), five died within the first year of life (median survival 0.5 years), and only one survived to 2.5 years (Case 11). This poor prognosis may be related to the location of p.Arg459 within the P-loop NTPase domain, a region critical for GTPase activity. Strikingly, this patient displayed a remarkable phenotypic specificity: despite harboring this severe mutation, the adrenal insufficiency was relatively mild, manifesting solely as pronounced skin hyperpigmentation while serum cortisol levels remained normal; nevertheless, the infant ultimately succumbed within the first year of life. Furthermore, the c.282T > G (p.Asn94Lys) variant is novel and has not been previously reported. Although the affected region is evolutionarily conserved, in silico analysis predicts a low probability of this variant disrupting protein structure or function. According to ACMG guidelines, it is classified as a variant of uncertain significance (VUS), and its pathogenicity awaits further functional validation. The co-occurrence of both c.1376G > A and c.282T > G mutations in this infant raises an important question for future research: whether these dual mutations exert a synergistic effect or partially counteract the toxicity of the p.Arg459Gln variant.

To better understand the clinical and molecular characteristics of this case, we performed a systematic literature review of previously reported cases of MIRAGE syndrome (Supplementary Table S2), The global distribution of MIRAGE syndrome shows the highest number of reported cases from Japan, the United States, and China. Most patients present with features including preterm birth, intrauterine and postnatal growth restriction, infections, enteropathy, adrenal insufficiency, and genital abnormalities (primarily cryptorchidism, hypospadias, and ambiguous genitalia). The majority of patients also exhibit myelodysplastic features such as thrombocytopenia and anemia, while a minority are diagnosed with MDS. The mutations c.3878G > A, c.1376G > A, and c.2471G > A have been identified as recurrent hotspot mutations. The mortality rate among patients is high (45%), with a young median age at death (1.09 years).

This study details a case of a preterm infant diagnosed with MIRAGE syndrome, manifesting respiratory distress, profound growth restriction, recurrent, severe infections, distinctive skin pigmentation, genital abnormalities, persistent feeding difficulties, thrombocytopenia, anemia, delayed white matter development, and ventriculomegaly. The infant also exhibited functional neurological impairments, including markedly diminished responsiveness and significant sucking-swallowing dysfunction, highlighting that the neurological prognosis of MIRAGE syndrome may be substantially underestimated and must be comprehensively communicated to families during genetic counseling. We further conducted a systematic review of the clinical and genetic phenotypic characteristics observed in infants under one year of age with this syndrome. Our findings significantly expand the known mutational spectrum of *SAMD9* and deepen the scientific understanding of the disorder's clinical and genetic landscape, offering significant clinical value for improved diagnosis and management strategies.

## Data Availability

The raw data supporting the conclusions of this article will be made available by the authors, without undue reservation.
