# Supplementary material for: Case Report: Unveiling the enigma: a rare male neonatal case of MIRAGE syndrome with female external genital presentation and literature review
Source: Front Pediatr. 2026 Jun 12;14:1808088. doi: 10.3389/fped.2026.1808088 (PMC13303219; doi:10.3389/fped.2026.1808088)
Supplement: Supplementary file 1 [file Table1.docx]

Supplementary Material

# Supplementary Table 1 Monitoring of Blood White Cells, Platelets, and Infection Markers

| **Content Items** | **1d** | **2d** | **4d** | **5d** | **10d** | **12d** | **17d** | **36d** | **38d** | **41d** | **46d** | **54d** | **57d** |
| --- | --- | --- | --- | --- | --- | --- | --- | --- | --- | --- | --- | --- | --- |
| WBC*10^9^/L | 14.82 | 11.78 | 7.03 | 11.20 | 10.09 | 9.77 | 10.66 | 11.25 | 15.61 | 10.46 | 13.38 | 8.45 | 9.8 |
| HGB g/L | 174 | 167 | 155 | 141 | 126 | 132 | 126 | 86 | 157 | 143 | 135.0 | 98.0 | 141 |
| PLT*10^9^/L | 66 | 36 | 24 | 123 | 91 | 90 | 117 | 160 | 119 | 101 | 103 | 140 | 122 |
| CRP mg/L |  | 3.66 |  | 11.37 | 104.80 | 40.26 | 4.03 | 21.32 | 61.81 | 8.69 |  |  |  |
| PCT ng/ml |  | 0.641 |  | 1.164 |  | 0.718 | 0.236 | 2.210 | 4.763 | 0.607 | 0.254 | 0.671 |  |

White blood cell count (WBC), Hemoglobin concentration(HGB), platelets (PLT), C - reactive protein (CRP), procalcitonin (PCT).

# Supplementary Table 2 Previously reported patients with MIRAGE syndrome carrying SAMD9 gene variants and their associated clinical phenotypes with neonatal/infantile onset.

| **case** | **References and Patient** | **Author/year** | **Country/Race** | **AAD(wk)** | **BW(g)** | **IUGR** | **Karyotype** | **G** | **I** | **E** | **A** | **PNGR** | **Thrombocytopenia** | **Anemia** | **M** | **SAMD9 variant** | **Outcome** |
| --- | --- | --- | --- | --- | --- | --- | --- | --- | --- | --- | --- | --- | --- | --- | --- | --- | --- |
| 1 | (4) | Li Yahui et al. /2022 | China | 30.14 | 900 | Yes | 46,XX | Normal | n.a. | n.a. | Yes | n.m. | Yes | n.m. | n.a. | c.4598G>A(p.R1533Q) | Died at 5 d due to respiratory and circulatory failure |
| 2 | (5)(p.1) | Zhu Lin et al.  /2021 | China | 36.86 | 1610 | Yes | 46,XY | Genital abnormalities hypospadias,bifid scrotum resembling labia | No | No | No | n.m. | Yes | n.m. | n.m. | c.2797A>G(p.I933V) | alive at 3mo |
| 3 | (5)(p.2) | Zhu Lin et al. /2021 | China | 31.71 | 1290 | Yes | 46,XY22pstk+ | Genital abnormalities hypospadias,cryptorchidism | Yes | Yes | No | n.m. | Yes | Yes | n.m. | c.2471G>A(p.R824Q) | alive at 8mo |
| 4 | (6) | Gu Jiali et al. /2022 | China | 31.71 | 1290 | Yes | 46,XY22pstk+ | Genital abnormalities hypospadias,micropenis,  testes in scrotum | Yes | Yes | n.m. | Yes | Yes | Yes | n.m. | c.2471G>A(p.R824Q) | alive |
| 5 | (7) | Zhu Ke et al./2024 | China | 36.00 | 1500 | n.m. | n.m. | Genital abnormalities penoscrotal transposition, chordee,hypospadias,  cryptorchidism | Yes | n.a. | No | Yes | n.m. | n.m. | Yes | c.3809T>A(p.F1270Y) | alive at 5yr and 1mo |
| 6 | (8) | He Xue et al./2024 | China | 35.00 | 1500 | Yes | 46,XY 22pstk+ | Genital abnormalities hypospadias,cryptorchidism,micropenis,scrotum resembling labia | Yes | Yes | No | Yes | Yes | No | n.m. | c.4598C>A(p.R1533Q) | alive at 5yr and 11mo |
| 7 | (9) | Zheng Rongfei et al./2021 | China | 31.71 | 1290 | n.m. | 46,XY 22pstk+ | Genital abnormalities incomplete masculinization, hypospadias,vaginal atresia,  small testes in scrotum | Yes | Yes | n.a. | Yes | Yes | Yes | n.m. | c.2471G>A(p.R824Q) | alive at 1yr and 5mo |
| 8 | (10) | Janjua et al./2022 | The United Arab Emirate | 34.71 | 1330 | Yes | 46,XY | Ambiguous genitalia, atrophic testes | Yes | Yes | Yes;  severe adrenal hypoplasia | n.m. | n.m. | n.m. | n.m. | c.3878G>A(p.R1293Q) | alive |
| 9 | (11) | Go et al./2023 | Korea | 29.86 | 656 | Yes | n.m. | Normal | Yes | Yes | Yes ;  adrenal hypoplasia | Yes | Yes | Yes | Yes | c.2483C>G(p.P828R) | alive at 15mo |
| 10 | (12) | Zhang Y et al. /2019 | China | 31.00 | 1000 | Yes | 46,XY | Genital abnormalities cryptorchidism | Yes | Yes | No | Yes | Yes | Yes | n.m. | c.2920 G>A(p.E974K) | Died at 12mo due to sepsis |
| 11 | (13) | Yoshizaki K et al. /2019 | Japan | 32.29 | 776 | Yes | 46,XX | Normal | Yes | Yes | Yes;  congenital adrenal hypoplasia | Yes | Yes | n.a. | n.a. | c.1376G＞A(p.R459Q) | alive at 2.5yr |
| 12 | (14)(p.1) | Sarthy et al./2018 | USA | 28.00 | n.m. | Yes | 46,XX | n.m. | Yes | Yes | Yes (Severe) | n.m. | Yes | Yes | n.a. | c.2462A>T(p.K821M) | Died at 23mo |
| 13 | (14)(p.2) | Sarthy et al./2018 | USA | 30.71 | 980 | Yes | 45,XY,-7[19]/XY[1] | Genital abnormalities micropenis,cryptorchidism | Yes | Yes | Yes (Severe) | n.m. | Yes | Yes | Yes (MDS) | c.2920G>A(p.E974K) | Died at 21mo |
| 14 | (15)(p.2) | Buonocore et al. /2017 | n.m. | 28.00 | 810 | Yes | 46,XY | Genital abnormalities cryptorchidism;female  genitalia | Yes | No | Yes(severe);adrenal agenesis | n.m. | Yes | Yes | n.m. | c.2945G>A(p.R982H) | Died at 1mo |
| 15 | (15)(p.3) | Buonocore et al. /2017 | n.m. | 34.00 | 1194 | Yes | 46,XY | Genital abnormalities cryptorchidism;female genitalia | Yes | Yes | Yes(severe);adrenal agenesis | n.m. | Yes | Yes | n.m. | c.2944C>T(p.R982C) | Died at 3mo |
| 16 | (15)(p.4) | Buonocore et al. /2017 | n.m. | 31.00 | 1160 | Yes | 46,XY | Genital abnormalities  cryptorchidism;female  genitalia | Yes | Yes | Yes(severe);adrenal agenesis | n.m. | Yes | Yes | n.m. | c.1376G>A(p.R459Q) | Died at 5mo |
| 17 | (15)(p.5) | Buonocore et al.  /2017 | n.m. | 31.00 | 880 | Yes | 46,XY | Genital abnormalities cryptorchidism;ambiguous genitalia | Yes | Yes | Yes(severe) | n.m. | No | No | n.m. | c.2944C>T(p.R982C) | Died at 9mo |
| 18 | (15)(p.6) | Buonocore et al./2017 | n.m. | 32.00 | 1150 | Yes | 46,XY | Genital abnormalities cryptorchidism;female  genitalia | Yes | Yes | Yes(severe) | n.m. | Yes | Yes | n.m. | c.2054G>A(p.R685Q) | Died at 21mo |
| 19 | (15)(p.7) | Buonocore et al.  /2017 | n.m. | 37.00 | 1680 | Yes | 46,XY | Genital abnormalitiesmale with severe hypospadias; descended testes | Yes | Yes | Yes(mild) | n.m. | Yes | No | Yes(MDS) | c.4707G>T(p.K1569N) | alive |
| 20 | (15)(p.8) | Buonocore et al./2017 | n.m. | 36.00 | 1860 | Yes | 46,XY | Genital abnormalities  cryptorchidism;female with  slight clitoromegaly | Yes | Yes | No | n.m. | Yes | No | Yes(MDS) | c.2948T>G(p.I983S) | alive |
| 21 | (16) | Kızılcan Çetin et al./2024 | Turkey | 39.00 | 2790 | n.m. | 45,XX,-7[45]/46,XX[5] | Genital abnormalities hypoplastic clitoris | n.m. | n.m. | No | Yes | Yes | No | No | c.2159del(p.N720TfsTer35) | alive at 6.5yr |
| 22 | (17)(p.1) | Jeffries et al./2017 | n.m. | 26.00 | n.m. | Yes | 46,XY | Genital abnormalitiesbifid scrotum,severe hypospadias, small penis,cryptorchidism; ambiguous genitalia | Yes | Yes | Yes | Yes | Yes | Yes | Yes | c.2471G>A(p.R824Q) | alive at 15mo |
| 23 | (17)(p.2) | Jeffries et al./2017 | n.m. | 32.00 | n.m. | Yes | 46,XY | Female genitalia;  intraabdominal testes removed | Yes | Yes | Yes | Yes | Yes | No | n.a. | c.3878G>A(p.R1293Q) | alive at 3.75yr |
| 24 | (18)(p.2) | Ahmed et al./2019 | Caucasian | 34.00 | 1425 | n.m. | n.m. | n.m. | Yes | Yes | Yes | Yes | Yes | Yes | Yes | c.4690G>A(p.G1564S) | alive at 5.2yr |
| 25 | (18)(p.5) | Ahmed et al./2019 | African American | 36.00 | 1895 | n.m. | n.m. | n.m. | Yes | Yes | n.m. | Yes | Yes | Yes | Yes | c.2407G>C(p.E803Q) | alive at 2.8yr |
| 26 | (19) | Chin et al./2021 | Singapore | 30.14 | 1145 | No | n.m. | Normal | Yes | n.a. | Yes;  adrenal hypoplasia | Yes | Yes | Yes | No | c.3406G>C(p.E1136Q) | alive at 2yr and 3mo |
| 27 | (20)(p.1) | Shima et al./2017 | Japan | n.m. | 1058 | Yes | 46,XY | Complete female genitalia | Yes | Yes | Yes;  bilateral adrenal agenesis | Yes(severe) | No | No | No | c.2053C>T(p.R685*)  and c.2165C>A(p.A722E) | alive at 8yr |
| 28 | (20)(p.2) | Shima et al./2017 | Germany | n.m. | 1870 | Yes | 46,XX | Normal | Yes | Yes | Yes | Yes | No | No | No | c.115C>T(p.Q39*) and  c.2306A>G(p.D769G) | Died at 12yr due to multiorgan failure from severe infection |
| 29 | (21) | Rentas et al./2020 | Caucasian | 28.00 | n.m. | Yes(severe) | 45,XY,−7 | Genital abnormalities hypospadias,cryptorchidism; ambiguous genitalia | Yes | Yes | yes | Yes | Yes | n.m. | Yes(MDS) | c.3221G>T(p.S1074I) | alive at 9yr |
| 30 | (22) | Fischer et al./2021 | Germany | n.m. | n.m. | n.m. | 46,XY | n.m. | n.m. | n.m. | n.m. | n.m. | n.m. | n.m. | n.m. | c.2054G>A(p.R685Q) | n.m. |
| 31 | (23) | Csillag et al./2018 | Austria | n.m. | n.m. | n.m. | n.m. | Genital abnormalities penoscrotal,hypospadias | Yes | Yes | Adrenal hypoplasia | n.m. | Yes | n.m. | Yes(mild) | c.1922T>C(p.L641P) | Died at 30mo due to respiratory failure |
| 32 | (24) | Basilious et al./2022 | Canada | 31.00 | 1170 | Yes | 46,XY | Genital abnormalities micropenis,hypospadias,  cryptorchidism;ambiguous genitalia | Yes | n.m. | Yes | Yes | Yes | Yes | n.m. | c.2945 G>A(p.R982H) | alive at 12mo |
| 33 | (25) | Cinleti et al./2023 | Turkey | 35.00 | 2150 | No | n.m. | n.m. | Yes | Yes | Yes | Yes | No | Yes | n.a. | c.3070A>G(p.K1024E) | alive at 10yr |
| 34 | (26) | Bove et al./2025 | USA | 36.00 | n.m. | Yes | 46,XY | Female genitalia | No | Yes | Yes;  adrenal atrophy | n.m. | Yes | Yes | n.m. | c.1376G>A(p.R459Q) | Died at 7mo |
| 35 | (27) | Saito et al./2023 | Japan | 32.43 | 911 | Yes | 46,XX | n.m. | n.m. | Yes | n.a. | Yes | n.a. | n.a. | n.m. | c.2945G>A(p.R982H) | alive at 236d |
| 36 | (1)(p1.1) | Narumi et al./2016 | Japan | n.m. | n.m. | Yes | 46,XY | Genital abnormalities | Yes | n.a. | Yes;  adrenal hypoplasia | n.a. | Yes | Yes | No | c.1376G>A(p.R459Q) | Died at 0.3yr |
| 37 | (1)(p2.1) | Narumi et al./2016 | Japan | n.m. | n.m. | Yes | 46,XX | Normal | Yes | Yes | Yes | Yes | Yes | n.a. | No | c.1376G>A(p.R459Q) | Died at 0.6yr |
| 38 | (1)(p3.1) | Narumi et al./2016 | Japan | n.m. | n.m. | Yes | 46,XY | Genital abnormalities | Yes | Yes | Yes | Yes | Yes | Yes | No | c.2305G>A(p.D769N) | alive at 16yr |
| 39 | (1)(p3.2) | Narumi et al./2016 | Japan | n.m. | n.m. | Yes | 46,XX | Normal | No | Yes | Yes | Yes | Yes | No | Yes(MDS) | c.2305G>A(p.D769N) | Died at 5yr due to MDS |
| 40 | (1)(p4.1) | Narumi et al./2016 | Japan | n.m. | n.m. | Yes | 46,XY | Genital abnormalities;  complete female genitalia | Yes | Yes | Yes | Yes | Yes | Yes | No | p.N834Y | Died at 1yr |
| 41 | (1)(p5.1) | Narumi et al./2016 | Japan | n.m. | n.m. | Yes | 46,XX | Normal | Yes | n.a. | Yes;  adrenal hypoplasia | n.a. | Yes | Yes | No | c.2920G>A(p.E974K) | Died at 0.1yr |
| 42 | (1)(p6.1) | Narumi et al./2016 | Japan | n.m. | n.m. | Yes | 46,XY | Genital abnormalities | Yes | Yes | Yes;  adrenal hypoplasia | Yes | Yes | Yes | No | p.A1195V | Died at 1yr |
| 43 | (1)(p7.1) | Narumi et al./2016 | Japan | n.m. | n.m. | Yes | 46,XY | Genital abnormalities | Yes | Yes | Yes;  adrenal hypoplasia | Yes | Yes | Yes | No | p.P1280L | alive at 1yr |
| 44 | (1)(p8.1) | Narumi et al./2016 | Japan | n.m. | n.m. | Yes | 46,XY | Genital abnormalities | Yes | Yes | Yes;  adrenal hypoplasia | Yes | Yes | Yes | No | p.Q1286K | alive at 12yr |
| 45 | (1)(p9.1) | Narumi et al./2016 | Japan | n.m. | n.m. | Yes | 46,XX | Normal | Yes | Yes | Yes | Yes | Yes | Yes | No | p.c.3878G>A(p.R1293Q) | Died at 2yr |
| 46 | (1)(p10.1) | Narumi et al./2016 | Japan | n.m. | n.m. | Yes | 46,XY | Genital abnormalities | Yes | Yes | Yes;  adrenal hypoplasia | Yes | Yes | Yes | Yes(MDS) | p.c.3878G>A(p.R1293Q) | Died at 2yr due to MDS |
| 47 | (28) | Shima H, et al.  /2018 | Japan | 30.00 | 834 | Yes | 46,XY | Complete female genital abnormalities hypospadias,  mild clitoromegaly | Yes | Yes | No | Yes | Yes | n.m. | n.m. | c.3049T>G( p.F1017V) | Died at 14mo due to hypovolemic shock |
| 48 | (29) | Formankova et al.  /2019 | Czechia | 32.43 | 1450 | n.m. | n.m. | Genital abnormalities hypospadias,  micropenis | Yes | Yes | No | n.m. | Yes | Yes | No | c.2471 G>A(p.R824Q) | Died at 440d due to sepsis |
| 49 | (30) | Roucher-Boulez et al./2019 | France | 36.71 | 1950 | Yes | 46,XY | Genital abnormalities  cryptorchidism,aurogenital sinus | n.m. | n.m. | No | n.a. | Yes | n.m. | No | c.2333C>T(p.(T778I) | alive at 14yr |
| 50 | (31) | Perisa et al./2019 | USA | 34.00 | 1843 | Yes | 46,XY | Genital abnormalities  hypospadias,cryptorchism | Yes | Yes | Yes | Yes(mild) | Yes | Yes | n.a. | c.2318T>C(p.l773T) | alive at 11yr |
| 51 | (32) | Buchbinder et al.  /2022 | USA | n.m. | n.m. | Yes | n.m. | Genital abnormalities  atrophic testis(left),  cryptorchidism(right) | Yes | Yes | n.m. | Yes | Yes | No | Yes(MDS) | c.1228T>G(p.Y410D) | n.m. |
| 52 | (33)(case1) | Viaene et al./2020 | USA | 30.86 | n.m. | n.m. | n.m. | Normal | Yes | Yes | Yes;  adrenal hypoplasia | No | n.m. | Yes | Yes | c.3878G>A(p.R1293Q) | Died |
| 53 | (33)(case2) | Viaene et al./2020 | USA | 28.00 | n.m. | n.m. | n.m. | Normal | Yes | No | Yes;  adrenal hypoplasia | Yes | Yes | n.m. | Yes | c.2462A>T(p.K821M) | Died |
| 54 | (34) | Ishiwa et al./2020 | Japan | 33.00 | 1064 | n.m. | n.m. | Normal | Yes | Yes | No | Yes | Yes | n.m. | No | c.4615 T>A(p.L1539I) | alive at 8yr |
| 55 | (35,36) | McDonald et al. /2010;  Wilson et al./2018 | Caucasian | 32.00 | 1157 | Yes | 46,XY | Genital abnormalities  microphallus,hypospadias,  and bilateral cryptorchidism | Yes | n.m. | Yes;  adrenal hypoplasia | Yes | n.m. | n.m. | Yes(MDS) | c.2691A>G(p.I897M) | alive at 6yr |
| 56 | (37) | Onuma et al./2020 | Japan | 31.00 | 912 | Yes | 46,XY | Ambiguous genitalia  micropenis,hypospadias,bifid scrotum | Yes | n.a. | Yes; adrenal hypoplasia | Yes | Yes | No | No | c.4435G>T(p.A1479S) | alive |
| 57 | (38) | Kawashima-Sonoyama et al. /2021 | Japan | 37.30 | 2002 | Yes | n.m. | n.m. | Yes | n.a. | No | Yes | Yes | Yes | Yes(MDS) | c.1310T>C(p.F437S) | Died at 6.33yr due to MDS |
| 58 | (2) | Mengen et al. /2020 | Turkey | 31.00 | 930 | Yes | 46,XY | Genital abnormalities  bilateral cryptorchidism | Yes | Yes | Yes; adrenal hypoplasia | Yes | Yes | Yes | n.m. | c.2920G>A(p.E974K) | Died due to multisystem organ failure |
| 59 | (39) | Kim et al. /2018 | Korea | 31.00 | 882 | Yes | 46,XY | Genital abnormalities micropenis,bilateral  cryptorchidism | Yes | n.m. | Yes; adrenal hypoplasia | n.m. | Yes | Yes | n.m. | c.2944C>T(p.R982C) | Died at 4mo due to septic shock |
| 60 | (40) | Baquedano-Lobera et al. /2021 | Spain | 31.00 | 900 | Yes | n.m. | Normal | Yes | n.m. | No | Yes | n.m. | n.m. | No | c.3878G>A(p.R1293Q) and c.2704C>T(p.R902W) | n.m. |
| 61 | (41) | Ishimori et al. /2025 | Japan | 35.00 | 1492 | n.a. | n.m. | Genital abnormalities  hypospadias | Yes | Yes | Yes | Yes | Yes | Yes | n.m. | c.3877C>T(p.R1293W) | Died at 21mo due to multi-organ failure |
| 62 | (42,43) | Peek et al./2025 | USA | 37.57 | 1901 | Yes | n.m. | Normal | Yes | Yes | Yes | Yes | Yes | n.a. | No | c.1972A>G(p.N658D) and c.88 C>T(p.Q30*) | alive at 5yr |
| 63 | (44) | Valdez  et al. /2024 | USA | 26.00 | n.m. | Yes | n.m. | Ambiguous genitalia | Yes | n.m. | Yes | n.m. | Yes | Yes | n.m. | n.m. | Died at 2yr |
| 64 | (45) | Niwinska-Faryna et al./2024 | Sweden | 34.00 | 1200 | n.m. | n.m. | n.a. | Yes | Yes | n.m. | Yes | n.m. | Yes | Yes | n.m. | alive at 14yr |
| 65 | (46) | Farakla et al./2024 | n.m. | 35.00 | 1410 | n.m. | 46,XY | Genital abnormalities  hypospadias and bifid scrotum | Yes | Yes | Yes; adrenal hypoplasia | Yes | n.m. | n.m. | No | c.137T>C(p.L46S) | alive at 17yr |
| 66 | (47) | Valencia et al. /2023 | USA | 30.00 | n.m. | Yes | n.m. | Normal | Yes | Yes | Yes | Yes | n.m. | n.m. | n.m. | n.m. | alive at 5mo |
| 67 | (48) | López et al./2023 | n.m. | n.m. | n.m. | Yes | 46,XY | n.m. | n.m. | n.m. | Adrenal hypoplasia | n.m. | n.m. | n.m. | n.m. | p.E646del | alive at 6mo |
| 68 | (49) | Furuzono et al. /2023 | Japan | n.m. | n.m. | n.m. | n.m. | Genital abnormalities  hypospadias and a bipartite scrotum | Yes | n.m. | Yes | Yes | n.m. | n.m. | n.m. | c.2305G>A(p.D769N) and p.C420R | alive at 24yr |
| 69 | (50) | Agarwal  /2022 | USA | 26.00 | n.m. | Yes | 46,XY | Ambiguous genitaliabifid scrotum and hypospadias | Yes | n.m. | Yes | n.m. | Yes | n.m. | n.m. | c.1457A>C(p.Y486S) | Died |
| 70 | (51) | Forbes et al./2020 | Australia | n.m. | n.m. | n.m. | n.m. | n.m. | n.a. | n.m. | n.m. | n.m. | Yes | Yes | Yes | c.4057A>G(p.K1353E) | alive at 7yr |

Age at delivery(AAD),Birth weight(BW),Intrauterine growth restriction (IUGR),**M**yelodysplastic **S**yndromes(MDS),Genitalia(G),Infection(I),Enteropathy(E),Adrenal insufficiency(A),Postnatal growth restriction(PNGR),Myelodysplasia(M);Day(d),Week(wk),month(mo),Year(yr);Patient.(p.),Not mentioned(n.m.),Not assessed (n.a.)
